# Cardiovascular Biomarkers and Diastolic Dysfunction in Patients With Chronic Chagas Cardiomyopathy

**DOI:** 10.3389/fcvm.2021.751415

**Published:** 2021-11-29

**Authors:** Luis E. Echeverría, Sergio Alejandro Gómez-Ochoa, Lyda Z. Rojas, Karen Andrea García-Rueda, Pedro López-Aldana, Taulant Muka, Carlos A. Morillo

**Affiliations:** ^1^Heart Failure and Heart Transplant Clinic, Fundación Cardiovascular de Colombia, Floridablanca, Colombia; ^2^Public Health and Epidemiological Studies Group, Cardiovascular Foundation of Colombia, Floridablanca, Colombia; ^3^Research Group and Development of Nursing Knowledge (GIDCEN-FCV), Research Center, Cardiovascular Foundation of Colombia, Floridablanca, Colombia; ^4^Department of Internal Medicine, Universidad de Antioquia, Medellín, Antioquia, Colombia; ^5^Institute of Social and Preventive Medicine (ISPM), University of Bern, Bern, Switzerland; ^6^Department of Cardiac Sciences, Libin Cardiovascular Institute of Alberta, Cumming School of Medicine, University of Calgary, Calgary, AB, Canada

**Keywords:** Chagas disease, Chagas cardiomyopathy, biomarkers, diastolic dysfunction (DD), echocardiograph

## Abstract

**Background:** Chronic Chagas Cardiomyopathy is a unique form of cardiomyopathy, with a significantly higher mortality risk than other heart failure etiologies. Diastolic dysfunction (DD) plays an important role in the prognosis of CCM; however, the value of serum biomarkers in identifying and stratifying DD has been poorly studied in this context. We aimed to analyze the correlation of six biochemical markers with diastolic function echocardiographic markers and DD diagnosis in patients with CCM.

**Methods:** Cross-sectional study of 100 adults with different stages of CCM. Serum concentrations of amino-terminal pro-B type natriuretic peptide (NT-proBNP), galectin-3 (Gal-3), neutrophil gelatinase-associated lipocalin (NGAL), high-sensitivity troponin T (hs-cTnT), soluble (sST2), and cystatin-C (Cys-c) were measured. Tissue Doppler imaging was used to measure echocardiographic parameters indicating DD. Multivariate logistic regression models adjusted by age, sex, BMI, and NYHA classification were used to evaluate the association between the biomarkers and DD.

**Results:** From the total patients included (55% male with a median age of 62 years), 38% had a preserved LVEF, but only 14% had a normal global longitudinal strain. Moreover, 64% had a diagnosis of diastolic dysfunction, with most of the patients showing a restrictive pattern (*n* = 28). The median levels of all biomarkers (except for sST2) were significantly higher in the group of patients with DD. Higher levels of natural log-transformed NTproBNP (per 1-unit increase, OR = 3.41, *p* < 0.001), Hs-cTnT (per 1-unit increase, OR = 3.24, *p* = 0.001), NGAL (per 1-unit increase, OR = 5.24, *p* =0.003), and Cys-C (per 1-unit increase, OR = 22.26, *p* = 0.008) were associated with increased odds of having diastolic dysfunction in the multivariate analyses. Finally, NT-proBNP had the highest AUC value (88.54) for discriminating DD presence.

**Conclusion:** Cardiovascular biomarkers represent valuable tools for diastolic dysfunction assessment in the context of CCM. Additional studies focusing mainly on patients with HFpEF are required to validate the performance of these cardiovascular biomarkers in CCM, allowing for an optimal assessment of this unique population.

## Introduction

Chagas disease, or American trypanosomiasis, is an infectious disease endemic in Latin America caused by hemoparasite *Trypanosomacruzi (T. cruzi)* (1). According to the World Health Organization (WHO) estimates published in 2015, 5,742,167 individuals with Chagas disease were living in Latin American countries (2). Moreover, estimated 300,000 infected individuals live in the U.S.A, and almost 100,000 live in the European Union with this disease, while the pooled prevalence of infection in Latin American migrants is estimated to be 4.2% (3, 4). The main transmission route of *T. cruzi* is vectorial (including parts or stool of ingestion triatomine insects); nevertheless, other important forms of the transmission include vertical (5), blood transfusions (6), and solid organ transplant (7). Although the acute infection is generally oligosymptomatic, almost 30% of the infected patients develop the chronic form of the disease after a few decades (8).

In its chronic phase, Chagas disease can involve the nervous system, the gastrointestinal tract (essentially the esophagus and the colon), and the heart (9). Chronic Chagas cardiomyopathy (CCM) represents one of the most severe forms of organ involvement, highlighting a high incidence of arrhythmias, leading to systemic embolisms and sudden cardiac death (10). Furthermore, it is associated with a severe myocardial involvement resulting in functional valvular regurgitation and dilated cardiomyopathy (11). Classically, heart failure in the context of Chagas disease has been related to a predominant systolic dysfunction profile (11, 12); however, diastolic dysfunction (DD) in the setting of CCM has also been associated with a worse clinical status and a higher incidence of adverse cardiovascular outcomes (13–16). This highlights the importance of making a prompt diagnosis of DD, which is usually achieved by echocardiography (17, 18).

Nevertheless, echocardiographic diagnosis of DD may suffer from the characteristic interobserver variability of this method (19). Moreover, patients with CD usually live in rural, secluded areas, significantly limiting the possibility of performing echocardiographic studies due to its restricted availability outside hospitals and associated costs (11). In this context, cardiovascular (CV) biomarkers could overcome these limitations, as samples can be collected and stored for a sufficient time to allow transportation to a nearby laboratory, the measurements do not depend on the availability of a trained cardiac sonographer, and the cost of the tests are significantly lower compared with echocardiography (20, 21). Moreover, CV biomarkers may also represent a viable option for identifying DD in this special population, as it has been observed that biomarkers such as NT-proBNP are significantly associated with DD diagnosis in heart failure of other etiologies (22–25). We aimed to analyze the correlation of six biochemical markers with diastolic function echocardiographic markers and DD diagnosis in patients with CCM. We hypothesize that NT-proBNP will have a significantly higher discriminative capacity to detect DD compared with the other CV biomarkers.

## Materials and Methods

### Study Population

This cross-sectional study was conducted between July and December 2015 at the Heart Failure and Heart Transplant Clinic of Fundación Cardiovascular in Floridablanca, Colombia. Adult outpatients (>18 years old) with a positive serological diagnosis of *T. cruzi* infection (positive IgG antibodies) and echocardiographic (echo) or electrocardiographic (ECG) abnormalities consistent with chronic Chagas cardiomyopathy (a left anterior fascicular block, a right bundle branch block, atrioventricular blocks, ventricular premature beats, atrial fibrillation or flutter, bradycardia ≤50 h/min or echocardiographic finding suggesting myocardial alterations as evaluated by a cardiologist) were included. We enrolled patients across all the severity stages, including individuals with implantable devices and refractory heart failure. The study sample was obtained from the patients with CCM, attending their follow-up evaluations; the first 100 individuals who fulfilled the inclusion criteria were enrolled. We excluded individuals with diabetes mellitus, coronary heart disease history, mitral stenosis, or uncontrolled hypertension. The Institutional Committee on Research Ethics approved the research protocol of the study. All the patients provided written informed consent for their participation in the study.

### Data Collection

Information on socioeconomic status, lifestyle factors, and medication use was recorded. Body-mass index, left ventricular ejection fraction (LVEF) calculated by Simpson's rule from the four-chamber view, and global longitudinal strain by speckle tracking (GLS) were also measured. Finally, fasting serum samples were collected from each individual for the assessment of the six biomarkers. High-sensitivity troponin T (hs-cTnT) was quantified with a fifth generation assay on an automated platform (ECLIA Elecsys 2010 analyzer, Roche Diagnostics, Germany). Galectin-3 (Gal-3) was assessed by using a quantitative method, specifically an ELFA (enzyme-linked fluorescent assay) technique (VIDAS, Biomerieux, Marcy l'Étoile, France). Amino-terminal pro-B-type natriuretic peptide (NT-proBNP) levels were measured using the electrochemiluminescence method (Roche Diagnostics GmbH, Mannheim, Germany). The Alere Triage^®^ NGAL test was used to assess Neutrophil Gelatinase-Associated Lipocalin (NGAL). Soluble ST2 (sST2) was measured from banked serum by a Critical Diagnostics Presage™ sST2 assay kit via enzyme-linked immunosorbent assay (ELISA). Finally, Cystatin c (Cys-c) was quantified by an immunologic turbid metric assay (Tina-quant Cystatin C cobas^®^).

### Echocardiography

Transthoracic echocardiography was performed using a GE Vivid S6 ultrasound system with an M4S matrix-array transducer of 1.6–4.3 MHz. Acquisitions were performed by a single certified and experienced cardiac sonographer blinded to the patient data (MCV). All echocardiograms were read and measured by a single cardiologist certified in echocardiography (LEE). Cardiac dimensions and Doppler measurements were obtained in accordance with American Society of Echocardiography and European Association of Echocardiography recommendations. M-mode echocardiography was used to measure the left atrial (LA) diameter and LV end-diastolic and end-systolic diameters (as recommended by the 2010 Guidelines of the American Society of Echocardiography at the time the study was initiated).

Two-dimensional LA and LV volumes were determined using modified Simpson's rule, with images obtained from the apical four-chamber and two-chamber views. Pulsed-wave Doppler was performed in the apical four-chamber view. From transmitral recordings, early peak (E) and late (A) diastolic filling velocities, E/A ratio, E wave deceleration time, velocity-time integral (VTI) of the E-wave (VTIE), A-wave VTI (VTIA), and LA filling fraction [VTIA/(VTIE + VTIA)] were obtained. The e' lateral velocity (tissue Doppler) and the E/e' ratio were calculated. Isovolumic relaxation time was measured from continuous-wave Doppler obtained in the apical five-chamber view. RV systolic pressure was derived from continuous-wave Doppler interrogation of tricuspid regurgitation. RV systolic function was evaluated by measuring the peak systolic myocardial velocity (RV S0) of the lateral tricuspid annulus, and tricuspid annular plane systolic excursion (TAPSE).

Regarding diastolic dysfunction, the patients were classified into four groups according to the echocardiographic characteristics observed. The patients without alterations compatible with DD were classified as having a normal filling pattern; moreover, individuals with DD were divided into three groups: abnormal relaxation pattern, pseudonormal pattern, and restrictive pattern (including both reversible and fixed defects) according to the recommendations for the evaluation of left ventricular diastolic function by echocardiography of the American Society of Echocardiography and the European Association of Cardiovascular Imaging.

### Statistical Analysis

Categorical variables were presented as numbers and proportions, while continuous variables were reported as medians and interquartile ranges. The Chi-square and Fischer exact tests were used to assess for differences in categorical variables by the DD group, while the Mann—Whitney *U*-test and the Kruskal—Wallis test were used for continuous variables. The Pearson's correlation test was used to assess whether the biomarkers were correlated with relevant continuous echocardiographic parameters of diastolic dysfunction. Furthermore, we used natural log-transformed values of biomarkers concentrations to approximate a normal distribution. ANOVA tests and the Bonferroni *post hoc* pairwise comparisons test were used to explore whether levels of natural log-transformed biomarkers were different across DD groups. Afterward, all biomarkers were analyzed using univariate logistic regression analyses in relation to DD diagnosis, and then adjusting for age, sex, body mass index, and NYHA classification. We quantified the discriminatory ability of the biomarkers with the area under the receiver-operating characteristic curve (AUC-ROC). The Youden index was used to identify the best biomarkers cut-off level to distinguish patients with and without DD. Also, we compared the AUC of NT-proBNP vs. all biomarkers. All statistical tests were two-sided. A *p*-value < 0.05 was considered statistically significant for all tests. All analyses were performed using Statistical Package STATA version 15 (Station College, Texas, USA).

### Sensitivity Analyses

To explore whether the associations of the levels of the biomarkers with the outcome of diastolic disfunction would differ by ejection fraction classification, an interaction term of each biomarker and the LVEF was tested. Furthermore, the performance of the biomarker was evaluated only in patients with a preserved ejection fraction to assess for differences that may have clinical implications in this context.

## Results

### Population Characteristics

One hundred individuals were included, 55% male with a median age of 62 [interquartile range (IQR) 53–70] years at baseline assessment. About 38% of the patients had a preserved LVEF, but only 14% of the included patients had a normal global longitudinal strain (GLS) value. Moreover, 64 patients (64%) had a diagnosis of diastolic dysfunction (DD), with most of the patients showing a restrictive pattern (*n* = 28). Patients with DD had a significantly higher body mass index (BMI) and were more frequently prescribed heart failure drugs, including ACEI/ARB, MRAs, beta-blockers, and diuretics, compared with patients without DD. Moreover, the individuals with DD had a significantly lower LVEF (Median in patients with DD: 17.5 vs. median in patients with non-DD: 42.5. *p* < 0.001) (Table 1).

**Table 1:** Characteristics of the patients with chronic Chagas cardiomyopathy evaluated according to diastolic dysfunction diagnosis (*n* = 100).

	**Patients without DD (*N* = 36)**	**Patients with DD (*N* = 64)**	**Total (*N* = 100)**	***p* value**
Males	18 (50.0%)	37 (57.8%)	55 (55.0%)	0.451
Age	60.5 (52.7, 71.0)	63.0 (56.0, 68.2)	62.0 (54.0, 69.5)	0.217
AHA/ACC Classification				0.066
A	1 (2.8%)	0 (0.0%)	1 (1.0%)	
B	9 (25.0%)	7 (10.9%)	16 (16.0%)	
C	26 (72.2%)	53 (82.8%)	79 (79.0%)	
D	0 (0.0%)	4 (6.2%)	4 (4.0%)	
NYHA				0.853
I	15 (41.7%)	28 (43.8%)	43 (43.0%)	
II	14 (38.9%)	25 (39.1%)	39 (39.0%)	
III	7 (19.4%)	10 (15.6%)	17 (17.0%)	
IV	0 (0.0%)	1 (1.6%)	1 (1.0%)	
BMI	27.3 (24.5, 29.7)	24.4 (21.9, 27.6)	26.2 (22.7, 28.2)	0.008
ACEI/ARB	16 (44.4%)	54 (84.4%)	70 (70%)	<0.001
MRA	9 (25.0%)	47 (73.4%)	56 (56%)	<0.001
Beta-blockers	19 (52.8%)	59 (92.2%)	78 (78%)	<0.001
Diuretics	10 (27.8%)	37 (57.8%)	47 (47.0%)	0.004
LVEF	42.5 (35.2, 46.2)	17.5 (8.7, 30.2)	27.000 (13.0, 40.2)	<0.001
NT-proBNP	132.0 (54.4, 319.6)	1695.0 (665.9, 3892.0)	703.6 (178.9, 2818.7)	<0.001
Hs-cTnT	5.1 (3.5, 11.2)	15.9 (9.5, 29.1)	11.7 (5.6, 22.4)	<0.001
Cys-C	1.0 (0.9, 1.1)	1.2 (1.0, 1.5)	1.1 (0.9, 1.4)	<0.001
NGAL	73.5 (61.2, 98.5)	110.0 (80.0, 160.2)	96.5 (69.0, 145.2)	<0.001
Galectin-3	12.9 (10.8, 15.1)	15.3 (12.2, 20.5)	14.2 (11.5, 18.2)	0.006
sST2	22.9 (19.4, 26.0)	26.7 (20.5, 33.5)	24.7 (20.1, 31.9)	0.091

*BMI, body mass index; ACEI/ARB, angiotensin-converting enzyme inhibitor/angiotensin receptor blocker; MRA, mineralocorticoid receptor antagonists; LVEF, left ventricular ejection fraction; NT-proBNP, N-terminal-proB-type natiuretic peptide; Hs-cTnT, high-sensitive cardiac troponin T; Cys-C, cystatin C; NGAL, neutrophil gelatinase- associated lipocalin; sST2, soluble ST2*.

#### Biomarkers and Diastolic Dysfunction in CCM

**Figure 1:**
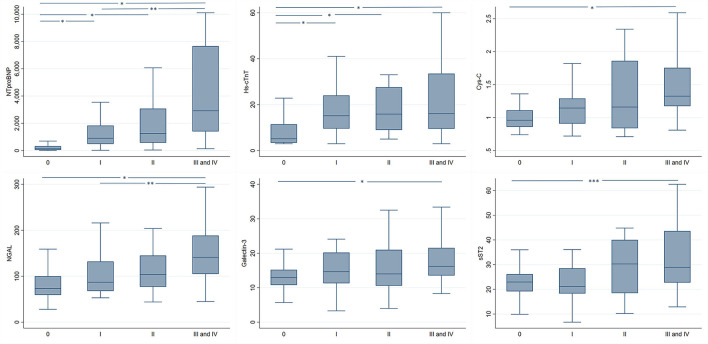
Biomarker levels in patients with chronic Chagas cardiomyopathy with normal and abnormal patterns of diastolic function of the left ventricle. NT-proBNP, N-terminal-proB-type natiuretic peptide; Hs-cTnT, high-sensitive cardiac troponin T; Cys-C, cystatin C; NGAL, neutrophil gelatinase-associated lipocalin; sST2, soluble ST2. *, p-value < 0.001. **, *p-*value < 0.01. ***, *p*-value < 0.05.

Significant differences in the levels of the biomarkers were observed when comparing patients with and without DD and across DD groups (Tables 1, 2). At first, the median levels of NT-proBNP (1,695 vs. 132 pg/ml), Hs-cTnT (15.9 vs. 5.1 ng/L), Cys-C (1.3 vs. 0.9 mg/L), NGAL (110 vs. 73.5 ng/ml), and Galectin-3 (15.3 vs. 12.9 ng/ml) were significantly higher in the group of patients with DD compared with those without this diagnosis. Similarly, the levels of the biomarker also increased with more severe forms of diastolic dysfunction, being significantly different across DD groups (Supplementary Table 1, Figure 1). Furthermore, most of the evaluated biomarkers were significantly correlated with diastolic function echocardiographic variables, being the NT-proBNP and the Cys-C the biomarkers with the highest correlation values across the assessed variables (Supplementary Table 2). Interestingly, Mitral flow E velocity was correlated only with the renal biomarkers (Cys-C and NGAL) (Supplementary Table 2).

**Table 2:** Characteristics of the patients with chronic Chagas cardiomyopathy evaluated according to diastolic dysfunction classification (*n* = 100).

	**0 (*N* = 36)**	**I (*N* = 22)**	**II (*N* = 14)**	**III and IV (*N* = 28)**	**Total (*N* = 100)**	***p* value**
Males	18 (50.0%)	12 (54.5%)	7 (50.0%)	18 (64.3%)	55 (55.0%)	0.686
Age	60.5 (52.7, 71.0)	62.0 (56.2, 67.7)	62.500 (55.0, 66.7)	64.500 (55.7, 69.7)	62.0 (54.0, 69.5)	0.660
**AHA/ACC Classification**						**0.019**
A	1 (2.8%)	0 (0.0%)	0 (0.0%)	0 (0.0%)	1 (1.0%)	
B	9 (25.0%)	2 (9.1%)	5 (35.7%)	0 (0.0%)	16 (16.0%)	
C	26 (72.2%)	20 (90.9%)	8 (57.1%)	25 (89.3%)	79 (79.0%)	
D	0 (0.0%)	0 (0.0%)	1 (7.1%)	3 (10.7%)	4 (4.0%)	
**NYHA**						0.650
I	15 (41.7%)	8 (36.4%)	8 (57.1%)	12 (42.9%)	43 (43.0%)	
II	14 (38.9%)	12 (54.5%)	4 (28.6%)	9 (32.1%)	39 (39.0%)	
III	7 (19.4%)	2 (9.1%)	2 (14.3%)	6 (21.4%)	17 (17.0%)	
IV	0 (0.0%)	0 (0.0%)	0 (0.0%)	1 (3.6%)	1 (1.0%)	
BMI	27.3 (24.5, 29.7)	27.2 (23.2, 28.0)	23.7 (21.4, 27.1)	23.1 (21.6, 27.5)	26.2 (22.7, 28.2)	**0.012**
ACEI/ARB	16 (44.4%)	17 (77.3%)	13 (92.9%)	24 (85.7%)	70 (70%)	**<0.001**
Beta-blockers	19 (52.8%)	21 (95.5%)	13 (92.9%)	25 (89.3%)	78 (78%)	**<0.001**
MRA	9 (25%)	16 (72.7%)	9 (64.3%)	22 (78.6%)	56 (56%)	**<0.001**
Diuretics	10 (27.8%)	11 (50.0%)	5 (35.7%)	21 (75.0%)	47 (47.0%)	**0.002**
LVEF	42.5 (35.2, 46.2)	26.5 (13.7, 32.5)	20.5 (10.0, 30.7)	14.0 (6.0, 21.2)	27.0 (13.0, 40.2)	**<0.001**

*BMI, body mass index; ACEI/ARB, angiotensin-converting enzyme inhibitor/angiotensin receptor blocker; MRA, mineralocorticoid receptor antagonists; LVEF, left ventricular ejection fraction. Bold values indicate a statistically significant difference, P-value < 0.05*.

Furthermore, multivariate logistic regression models adjusted by age, sex, BMI, and NYHA classification revealed that all logarithm-transformed biomarkers (except for Galectin-3 and sST2) were significantly associated with DD diagnosis (Table 3). In this context, higher levels of natural log-transformed NTproBNP (per 1-unit increase, OR = 3.41, *p* < 0.001), Hs-cTnT (per 1-unit increase, OR = 3.24, *p* = 0.001), NGAL (per 1-unit increase, OR = 5.24, *p* = 0.003), and Cys-C (per 1-unit increase, OR = 22.26, *p* = 0.008) were associated with increased odds of having diastolic dysfunction.

**Table 3:** Association between biomarker levels and diastolic dysfunction diagnosis in patients with chronic Chagas cardiomyopathy.

**Biomarkers**	**Crude estimate**			**Adjusted estimate^Ω^**		
	**OR**	**95% CI**	***p*-value**	**OR**	**95% CI**	***p*-value**
NT-proBNP	2.54	1.75–3.67	**<0.001**	3.41	2.02–5.74	**<0.001**
Hs-cTnT	3.41	1.89–6.20	**<0.001**	3.24	1.65–6.37	**0.001**
Cys-C	18.25	3.16–105.38	**0.001**	22.26	2.27–217.7	**0.008**
NGAL	6.42	2.26–18.28	**<0.001**	5.24	1.74–15.78	**0.003**
Galectin-3	3.73	1.20–11.55	**0.023**	2.89	0.87–9.57	0.081
sST2	1.87	0.81–4.32	0.142	1.56	0.63–3.88	0.339

Ω *, All models were adjusted by age, sex, body mass index, and NYHA classification. NT-proBNP, N-terminal-proB-type natiuretic peptide; Hs-cTnT, high-sensitive cardiac troponin T; Cys-C, cystatin C; NGAL, neutrophil gelatinase-associated lipocalin; sST2, soluble ST2. Bold values indicate a statistically significant difference, P-value < 0.05*.

#### Diagnostic Evaluation of the Biomarkers

Table 4 shows the best cut-off point for each biomarker and its corresponding AUC-ROC for detecting diastolic dysfunction. NT-proBNP had the highest AUC value (88.54) and was superior in all evaluated parameters (sensitivity, specificity, positive predictive value, negative predictive value, and accuracy) than the other assessed biomarkers. Hs-cTnT and Cystatin-C had the second and third highest AUC values, respectively. Finally, Galectin-3 was the only biomarker that was not significantly associated with DD diagnosis.

**Table 4:** Discriminative characteristics of the evaluated biomarkers for DD in the context of chronic Chagas cardiomyopathy.

**Biomarkers^Ω^**	**Cut-off**	**AUC (%)**	**Sensitivity (%)**	**Specificity (%)**	**PPV (%)**	**NPV (%)**	**Accuracy (%)**	**p-value**
NT-proBNP	5.81	88.54	90.63	77.78	87.88	82.35	86	<0.001
Hs-cTnT	2.30	77.30	79.69	66.67	80.95	64.86	75	<0.001
Cystatin-C	0.07	76.22	81.25	50	74.29	60	70	0.002
NGAL	4.48	73.22	79.69	44.44	71.83	55.17	67	0.010
sST2	3.27	72.09	82.81	41.67	71.62	57.69	68	0.066
Galectin-3	2.42	69.57	85.94	33.33	69.62	57.14	67	0.218

Ω *, The discriminative capacity of the biomarkers was evaluated, considering the models adjusted by age, sex, body mass index, and NYHA classification. NT-proBNP, N-terminal-proB-type natiuretic peptide; Hs-cTnT, high-sensitive cardiac troponin T; Cys-C, cystatin C; NGAL, neutrophil gelatinase-associated lipocalin; sST2, soluble ST2*.

**Figure 2:**
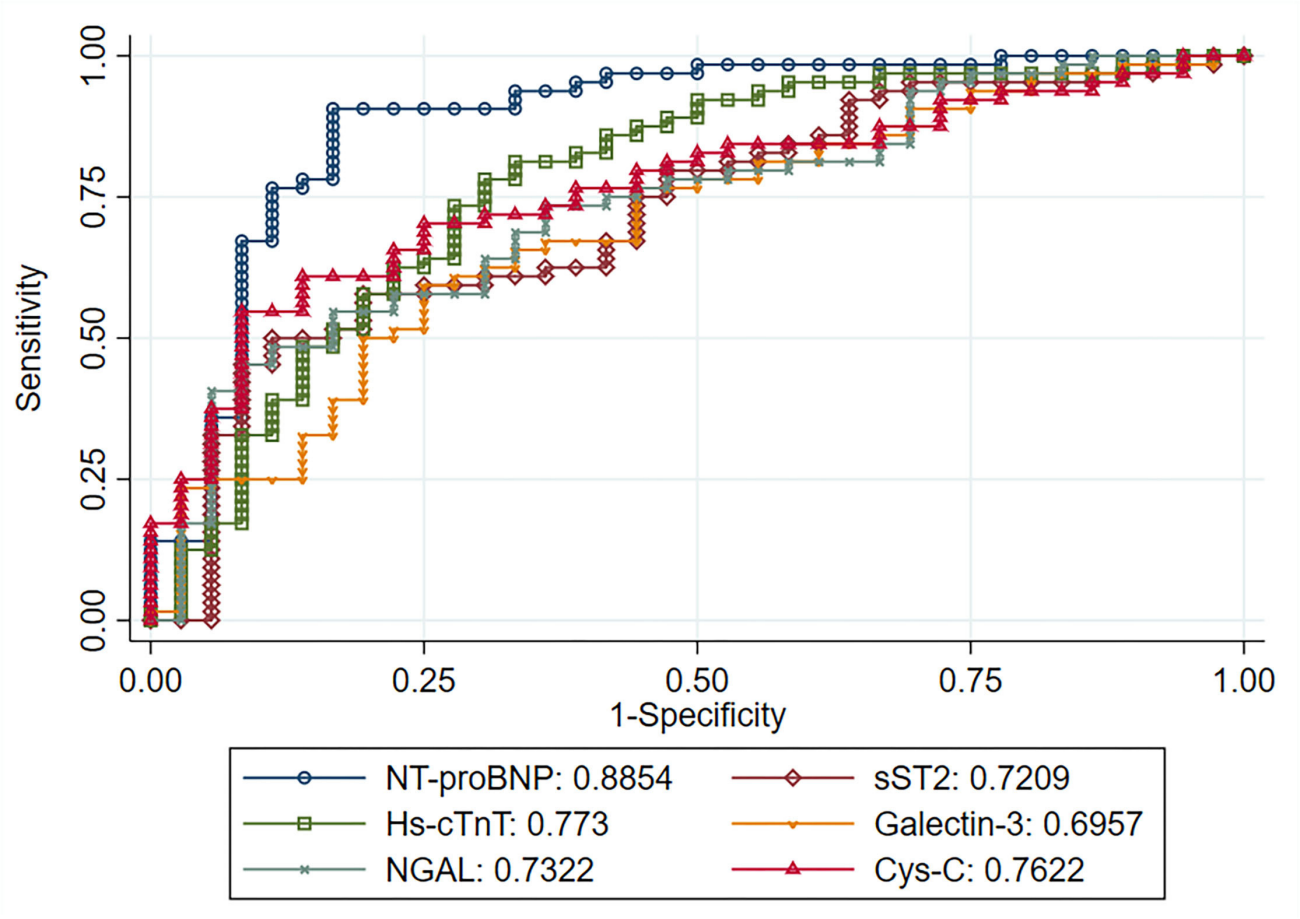
An area under the curve of each natural log-transformed biomarker regarding the ability of discriminating patients with diastolic dysfunction. NT-proBNP, N-terminal-proB-type natiuretic peptide; Hs-cTnT, high-sensitive cardiac troponin T; Cys-C, cystatin C; NGAL, neutrophil gelatinase-associated lipocalin; sST2, soluble ST2.

When comparing the AUC-ROC, we observed that the value was significantly higher for the NT-proBNP compared with any of the other biomarkers (*p*: 0.015 vs. Hs-cTnT, p: 0.004 vs. sST2, *p* < 0.001 vs. Galectin-3, *p*: 0.004 vs. NGAL, p: 0.010 vs. Cys-C) (Figure 2).

### Sensitivity Analyses

A significant interaction term by LVEF was observed for NT-proBNP (*p* = 0.011) and Hs-cTnT (*p* = 0.021). Therefore, we assessed the population of patients with preserved ejection fraction apart, as identifying diastolic dysfunction could be more challenging in this subgroup. Supplementary Table 3 summarizes the characteristics of patients with HF with a preserved ejection fraction according to the diagnosis of DD, evidencing a similar profile to the one observed in the full cohort (Table 1). We observed a different performance of the biomarkers in this population, highlighting that NT-proBNP and Hs-cTnT remained as significant predictors of DD. At the same time, NGAL and Cys-C lost statistical significance, and Galectin-3 and sST2 remained not significantly associated with diastolic dysfunction diagnosis (Supplementary Table 4).

## Discussion

In this study, patients with chronic Chagas cardiomyopathy had high prevalence of DD (64%), with most of the patients showing a restrictive pattern (*n* = 28). Several differences were observed between the patients with and without DD, highlighting a significantly lower LVEF in patients with DD diagnosis. Interestingly, patients with diastolic dysfunction had a significantly lower body mass index compared with those without DD. This finding could reflect the most advanced stage of HF in patients with DD, as these individuals develop a syndrome of cardiac cachexia, which is characterized by significant weight loss in the absence of peripheral edema. Furthermore, significant differences in the levels of NT-proBNP, Hs-cTnT, Cys-C, NGAL, and Galectin-3 were observed when comparing patients with and without DD and across DD groups, which were evidenced. Finally, NT-proBNP had the highest discriminative capacity to identify patients with DD, being its AUC-ROC significantly higher than those of the other assessed biomarkers.

Diastolic dysfunction has been associated with the development of Chagas cardiomyopathy in several studies, highlighting the relevant role of this condition as a potential early marker of myocardial involvement in the context of Chagas disease (13, 14, 17, 26). In the study of García-Alvarez et al., half of the patients initially classified as being in the undetermined form of the disease, which showed impaired relaxation patterns. In contrast, half of the individuals with electrocardiographic abnormalities suggestive of CCM had a normal diastolic function (13). Furthermore, DD has also been associated with higher mortality risk among patients with CCM, as Rassi et al. suggested in their study, concluding that left atrial volume predicts adverse cardiovascular outcomes in this context (12). Similarly, Nunes et al. observed a significant association between the E/e' ratio and a composite outcome of mortality and heart transplantation (27). Nevertheless, a significant interaction between the LVEF and the E/e' ratio was observed, as, in patients with a severe LV systolic function (LVEF <30%), the E/e' ratio had an inverse association with mortality; on the other hand, in patients with LVEF >45%, the E/e' ratio had a direct association with mortality, being a ratio >15 a powerful predictor of the assessed outcome. Interestingly, the septal e' velocity in patients with the composite outcome was >7 cm/sg on average. This observation deserves attention and, probably, an in-depth study of confounding factors that could explain it (27).

On the other hand, serum biomarkers have been gaining relevance as an important tool in the context of CCM. Several studies have highlighted the usefulness of these measurements to detect, classify, and assess the prognosis of patients with Chagas disease and myocardial involvement (28, 29). Among the most relevant ones, NT-proBNP has been the biomarker with the best performance to detect and predict adverse outcomes in patients with CCM, showing an unparalleled discriminative capacity compared with other biomarkers (30–33). Nevertheless, few studies have assessed performance of this biomarker in detecting and classifying DD in CD. Barbosa et al. reported that NT-proBNP levels were significantly associated with diastolic dysfunction in 59 patients with dilated CCM in Brazil. They observed that a marked elevated concentration of this biomarker (>800 pg/ml) had a sensitivity of 90% and specificity of 70.5% for detecting a restrictive filling pattern in this population (14). Similarly, Mady et al. reported that NT-proBNP levels were significantly correlated with DD echocardiographic markers (26). Finally, the study of Nunes MCP et al. concluded that BNP levels were significantly correlated with diastolic function patterns independently of systolic function in a small cohort of patients with CCM. A BNP value of 280.4 pg/ml showed a sensitivity of 96% and a specificity of 75% for predicting a value of E/e' ratio > 15, while the AUC-ROC for BNP to detect an E/e' ratio > 15 was 0.875 (34).

Although several studies have highlighted the potential use of biomarkers as Galectin-3, Cystatin-C, and NGAL to identify DD in patients with different diseases, this represents the first study that assessed these and other relevant cardiovascular biomarkers in the context of CD and CCM (22, 25, 35). Furthermore, NT-proBNP levels are mainly elevated by systolic dysfunction alone, potentially limiting its utility in the context of HF with a preserved ejection fraction (HFpEF) and patients with mild DD (14). Considering this limitation, additional cardiorenal biomarkers could prove useful in this specific context. Our findings of a significant association between Hs-cTnT, Cys-C, NGAL, and Galectin-3 levels with diastolic dysfunction provide valuable evidence regarding the role of different pathophysiological pathways, reflected in the differential levels of these biomarkers, in the development of diastolic dysfunction in CCM. However, despite showing a statistically significant difference, the difference in the levels of Cys-C in patients with DD compared with those without this condition may not have a relevant clinical significance. Moreover, we also observed that some biomarkers showed a higher correlation with specific echocardiographic markers of DD than NT-proBNP. For example, NGAL had an importantly higher correlation with PSAP (0.53) and the E/A ratio (0.35) compared to NT-proBNP (0.34 and 0.24, respectively). Similarly, Cys-C had a higher correlation with PSAP (0.45) and the E/e' lateral ratio (0.44) compared to NT-proBNP (0.34 and 0.22, respectively). Interestingly, NT-proBNP was not significantly correlated with the mitral flow E velocity, being this measure only correlated with the biomarkers of renal impairment (Cys-C and NGAL).

Finally, it is relevant to highlight that biomarkers are not intended to replace echocardiography regarding DD diagnosis; however, they could be of high utility in settings with limited access to echocardiography by identifying those patients with early CCM with a high risk of functional derangements of the LV, thus optimizing the use of the echocardiographic assessment for only this high-risk group. Nevertheless, the relevance and added value of cardiorenal biomarkers in addition to NT-proBNP in CCM still need to be evaluated in larger cohorts. In addition, the comparative performance of these biomarkers to detect DD in patients with HF of other etiologies is also warranted, as the present results cannot be extrapolated to cardiomyopathies other than CCM due to its unique pathophysiology and patterns of myocardial involvement.

### Study Limitations

We must acknowledge the important limitations of our study. At first, diastolic dysfunction diagnosis was not achieved through cardiac catheterization, which is currently considered the gold standard for this purpose. Secondly, the median LVEF was significantly lower in patients in the diastolic dysfunction group than in individuals without DD. It is critical to consider this finding when interpreting the results of our study, as the high prevalence of reduced LVEF in patients with DD from our population can significantly modify the associations observed. We aimed to overcome this limitation by performing a sensitivity analysis, assessing the performance of the biomarkers separately in patients with preserved LVEF (>40%), observing similar findings for NT-proBNP and Hs-cTnT. However, future studies with larger sample sizes are required to validate these results, as our sample did not include an important number of patients with a preserved ejection fraction; therefore, we could not thoroughly assess the performance of the biomarkers in patients with diastolic dysfunction and preserved LVEF. In addition, a control group of patients with other cardiomyopathies was not included, which precluded the comparison of the discriminative capacity of the biomarkers in non-CCM HF. Finally, we did not measure the intraobserver variability and the coefficient of variation for the assessed biomarkers, limiting the possibility of considering these relevant variables in our analyses.

## Conclusions

Cardiovascular biomarkers represent valuable tools for diastolic dysfunction assessment in the context of CCM. In the present study, NT-proBNP showed the highest discriminative capacity for detecting DD; nevertheless, due to limitations of its use in the setting of reduced LVEF, other biomarkers, such as Hs-cTnT, Cys-C, NGAL, and Galectin-3, could prove useful for detecting and classifying DD when echocardiography or other diagnostic methods are not available. Additional studies focusing mainly on patients with HFpEF are required to validate the performance of cardiovascular biomarkers in CCM, allowing for an optimal assessment of this special population.

## Data Availability Statement

The datasets presented in this article are not readily available due to restrictions from the author's host institution. Requests to access the datasets should be directed to Luis Eduardo Echeverría, luisedo10@gmail.com.

## Ethics Statement

The studies involving human participants were reviewed and approved by Comité de Ética en Investigación de la Fundación Cardiovascular de Colombia. The patients/participants provided their written informed consent to participate in this study.

## Author Contributions

LE, LR, TM, and CM contributed to the conception and design of the study. SG-O, KG-R, and PL-A organized the database. SG-O, LR, and TM performed the statistical analysis. SG-O wrote the first draft of the manuscript. SG-O, LE, LR, KG-R, PL-A, and CM wrote sections of the manuscript. All authors contributed to manuscript revision, read, and approved the submitted version.

## Funding

LR and TM were supported by St. Gallen University through the Seed Money Grants Call of 2019. LE and LR were also supported by the Colombian government through Departamento Administrativo de Ciencia, Tecnología e Innovación-COLCIENCIAS (project code 501453730398, CT 380–2011); http://www.colciencias.gov.co/. The funder had no role in study design, data collection, analysis, decision to publish, or manuscript preparation.

## Conflict of Interest

The authors declare that the research was conducted in the absence of any commercial or financial relationships that could be construed as a potential conflict of interest.

## Publisher's Note

All claims expressed in this article are solely those of the authors and do not necessarily represent those of their affiliated organizations, or those of the publisher, the editors and the reviewers. Any product that may be evaluated in this article, or claim that may be made by its manufacturer, is not guaranteed or endorsed by the publisher.
