# Supplementary material for: Cardiovascular Biomarkers and Diastolic Dysfunction in Patients With Chronic Chagas Cardiomyopathy
Source: Front Cardiovasc Med. 2021 Nov 29;8:751415. doi: 10.3389/fcvm.2021.751415 (PMC8666535; doi:10.3389/fcvm.2021.751415)
Supplement: Supplementary file 1 [file Data_Sheet_1.docx]

**Cardiovascular Biomarkers and Diastolic Dysfunction in Patients with Chronic Chagas Cardiomyopathy**

**Supplementary material**

**Supplementary Table 1.** Biomarkers levels across diastolic dysfunction categories in patients with chronic Chagas cardiomyopathy.

| Variable | 0 | I | II | III-IV | Total | p-value |
| --- | --- | --- | --- | --- | --- | --- |
| NT-proBNP | 132.0 (54.4, 319.6) | 897.9 (505.4, 1832.2) | 1246.0 (610.3, 3042.2) | 2914.5 (1435.5, 7595.5) | 703.6 (178.9, 2818.7) | 0 vs I: 0.001; 0 vs II: 0.001; 0 vs III-IV: <0.001; I vs II: 1.000; I vs III: 0.037; II vs III: 0.274. |
| Hs-cTnT | 5.1 (3.5, 11.2) | 15.2 (9.7, 23.9) | 15.9 (9.6, 26.2) | 16.0 (9.7, 33.2) | 11.7 (5.6, 22.4) | 0 vs I: 0.001; 0 vs II: 0.005; 0 vs III-IV: <0.001; I vs II: 1.000; I vs III: 1.000; II vs III: 1.000. |
| Cys-C | 1.0 (0.9, 1.1) | 1.1 (0.9, 1.3) | 1.2 (0.9, 1.7) | 1.3 (1.2, 1.7) | 1.1 (0.9, 1.4) | 0 vs I: 0.344; 0 vs II: 0.189; 0 vs III-IV: <0.001; I vs II: 1.000; I vs III: 0.079; II vs III: 0.445. |
| NGAL | 73.5 (61.2, 98.5) | 87.0 (68.2, 127.0) | 104.0 (78.0, 142.0) | 141.0 (105.5, 179.7) | 96.5 (69.0, 145.2) | 0 vs I: 0.437; 0 vs II: 0.139; 0 vs III-IV: <0.001; I vs II: 1.000; I vs III: 0.007; II vs III: 0.168. |
| Galectin-3 | 12.9 (10.8, 15.1) | 14.6 (11.4, 19.8) | 14.0 (10.8, 20.2) | 16.2 (13.6, 20.9) | 14.2 (11.5, 18.2) | 0 vs I: 0.331; 0 vs II: 0.854; 0 vs III-IV: 0.005; I vs II: 1.000; I vs III: 0.620; II vs III: 0.493. |
| sST2 | 22.9 (19.4, 26.0) | 21.2 (18.8, 28.1) | 30.2 (19.0, 38.6) | 28.9 (23.1, 40.5) | 24.7 (20.1, 31.9) | 0 vs I: 1.000; 0 vs II: 0.615; 0 vs III-IV: 0.033; I vs II: 0.589; I vs III: 0.050; II vs III: 1.000. |

**Supplementary Table 2.** Pearson's correlation coefficient between biomarkers levels and relevant echocardiographic variables of diastolic dysfunction in patients with chronic Chagas cardiomyopathy.

|  | NT-proBNP | p-value | Hs-cTnT | p-value | Cys-C | p-value | NGAL | p-value | Galectin-3 | p-value | sST2 | p-value |
| --- | --- | --- | --- | --- | --- | --- | --- | --- | --- | --- | --- | --- |
| LA volume index (mL/m2) | 0.3935 | **<0.001** | 0.2560 | **<0.001** | 0.3972 | **<0.001** | 0.3579 | **<0.001** | 0.4151 | **<0.001** | 0.3557 | **<0.001** |
| PSAP (mmHg) | 0.3436 | **<0.001** | 0.0696 | 0.5169 | 0.4533 | **<0.001** | 0.5261 | **<0.001** | 0.2796 | **0.008** | 0.2786 | **0.0082** |
| Mitral flow E velocity (cm/s) | 0.0000 | 0.9997 | -0.1121 | 0.2718 | 0.2120 | **0.0361** | 0.2828 | **0.0048** | 0.0718 | 0.4825 | 0.0342 | 0.7381 |
| E/A ratio | 0.2389 | **0.0241** | 0.0421 | 0.6949 | 0.2882 | **0.0062** | 0.3523 | **<0.001** | 0.1037 | 0.3336 | 0.0768 | 0.4745 |
| S/D ratio | -0.5513 | **0.0078** | 0.3954 | 0.0686 | -0.4960 | **0.0189** | -0.2073 | 0.3546 | -0.5079 | **0.0158** | -0.2177 | 0.3304 |
| e' lateral velocity (cm/s) | -0.2122 | **0.0446** | -0.2463 | **0.0193** | -0.2601 | **0.0133** | -0.2184 | **0.0386** | -0.2757 | **0.0085** | -0.1576 | 0.1379 |
| E/e' lateral ratio | 0.2213 | **0.0394** | 0.0957 | 0.3780 | 0.4431 | **<0.001** | 0.3706 | **0.0004** | 0.3070 | **0.0038** | 0.2048 | 0.0570 |
| LV end-diastolic volume (ml) | 0.5489 | **0.0030** | 0.5132 | **0.0062** | 0.4155 | **0.0311** | 0.3175 | 0.1065 | 0.2920 | 0.1394 | 0.3310 | 0.0917 |
| LV mass index (g/m2) | 0.6188 | **<0.001** | 0.4770 | **<0.001** | 0.3840 | **<0.001** | 0.3758 | **<0.001** | 0.4341 | **<0.001** | 0.3373 | **<0.001** |

**Supplementary Table 3**. Characteristics of the patients with Chronic Chagas Cardiomyopathy and a preserved ejection fraction evaluated according to diastolic dysfunction diagnosis (n=59).

|  | Patients without DD (N=33) | Patients with DD (N=26) | Total (N=59) | p value |
| --- | --- | --- | --- | --- |
| Males | 17 (51.5%) | 14 (53.8%) | 31 (52.5%) | 0.859 |
| Age | 59.000 (52.000, 64.000) | 62.500 (56.250, 67.750) | 60.000 (53.500, 67.000) | 0.140 |
| AHA/ACC Classification |  |  |  | 0.609 |
| A | 1 (3.0%) | 0 (0.0%) | 1 (1.7%) |  |
| B | 9 (27.3%) | 6 (23.1%) | 15 (25.4%) |  |
| C | 23 (69.7%) | 20 (76.9%) | 43 (72.9%) |  |
| NYHA |  |  |  | 0.902 |
| 1 | 15 (45.5%) | 13 (50.0%) | 28 (47.5%) |  |
| 2 | 13 (39.4%) | 10 (38.5%) | 23 (39.0%) |  |
| 3 | 5 (15.2%) | 3 (11.5%) | 8 (13.6%) |  |
| IMC | 27.398 (24.623, 29.903) | 26.643 (23.102, 27.728) | 27.099 (23.926, 29.158) | 0.237 |
| ACEI/ARB | 14 (42.4%) | 20 (76.9%) | 34 (57.6%) | 0.008 |
| MRA | 6 (18.2%) | 15 (57.7%) | 21 (35.6%) | 0.002 |
| Beta-blockers | 16 (48.5%) | 22 (84.6%) | 38 (64.4%) | 0.004 |
| Diuretics | 7 (21.2%) | 11 (42.3%) | 18 (30.5%) | 0.081 |
| LVEF | 60.000 (52.000, 63.000) | 49.500 (43.250, 54.750) | 55.000 (46.500, 61.000) | < 0.001 |
| NT-proBNP | 113.300 (50.530, 238.300) | 659.400 (340.675, 1431.250) | 238.300 (66.380, 659.400) | < 0.001 |
| NGAL | 71.000 (56.000, 92.000) | 82.000 (63.500, 110.000) | 74.000 (61.000, 102.500) | 0.151 |
| Cys-C | 0.940 (0.860, 1.060) | 1.060 (0.840, 1.318) | 0.960 (0.845, 1.150) | 0.268 |
| Galectin-3 | 12.500 (10.800, 14.800) | 13.650 (11.375, 17.750) | 13.400 (10.800, 15.550) | 0.310 |
| sST2 | 22.900 (19.600, 25.400) | 21.200 (17.175, 27.050) | 22.100 (18.600, 26.150) | 0.598 |
| Hs-cTnT | 4.750 (3.130, 8.980) | 12.710 (7.203, 23.027) | 7.000 (3.880, 14.000) | 0.002 |

**Supplementary Table 4.** Discriminative characteristics of the evaluated biomarkers for DD in the context of patients with Chronic Chagas Cardiomyopathy and a preserved ejection fraction.

| Biomarkers^Ω^ | Cut-off | AUC (%) | Sensitivity (%) | Specificity (%) | PPV (%) | NPV (%) | Accuracy (%) | p-value |
| --- | --- | --- | --- | --- | --- | --- | --- | --- |
| *NT-proBNP* | 5.81 | 85.78 | 76.92 | 84.85 | 80 | 82.35 | 81.36 | <0.001 |
| *Hs-cTnT* | 2.30 | 75.76 | 61.54 | 78.79 | 69.57 | 72.22 | 71.19 | 0.009 |
| *Cystatin-C* | 0.07 | 65.27 | 42.31 | 75.76 | 57.89 | 62.50 | 61.02 | 0.503 |
| *NGAL* | 4.48 | 69.46 | 46.15 | 72.73 | 57.14 | 63.16 | 61.02 | 0.119 |
| *sST2* | 3.27 | 65.73 | 34.62 | 72.73 | 50 | 58.54 | 55.93 | 0.456 |
| *Galectin-3* | 2.42 | 64.80 | 42.31 | 63.64 | 47.83 | 58.33 | 54.24 | 0.566 |

^Ω^: The discriminative capacity of the biomarkers was evaluated considering the models adjusted by age, sex, body mass index and NYHA classification.
